# Double Loading of Breast Implants in Aesthetic and Reconstructive Plastic Surgery With the iNPLANT Funnel

**DOI:** 10.1093/asjof/ojab012

**Published:** 2021-03-12

**Authors:** Paul Rosenberg, Luis Rios

**Figure 1.**
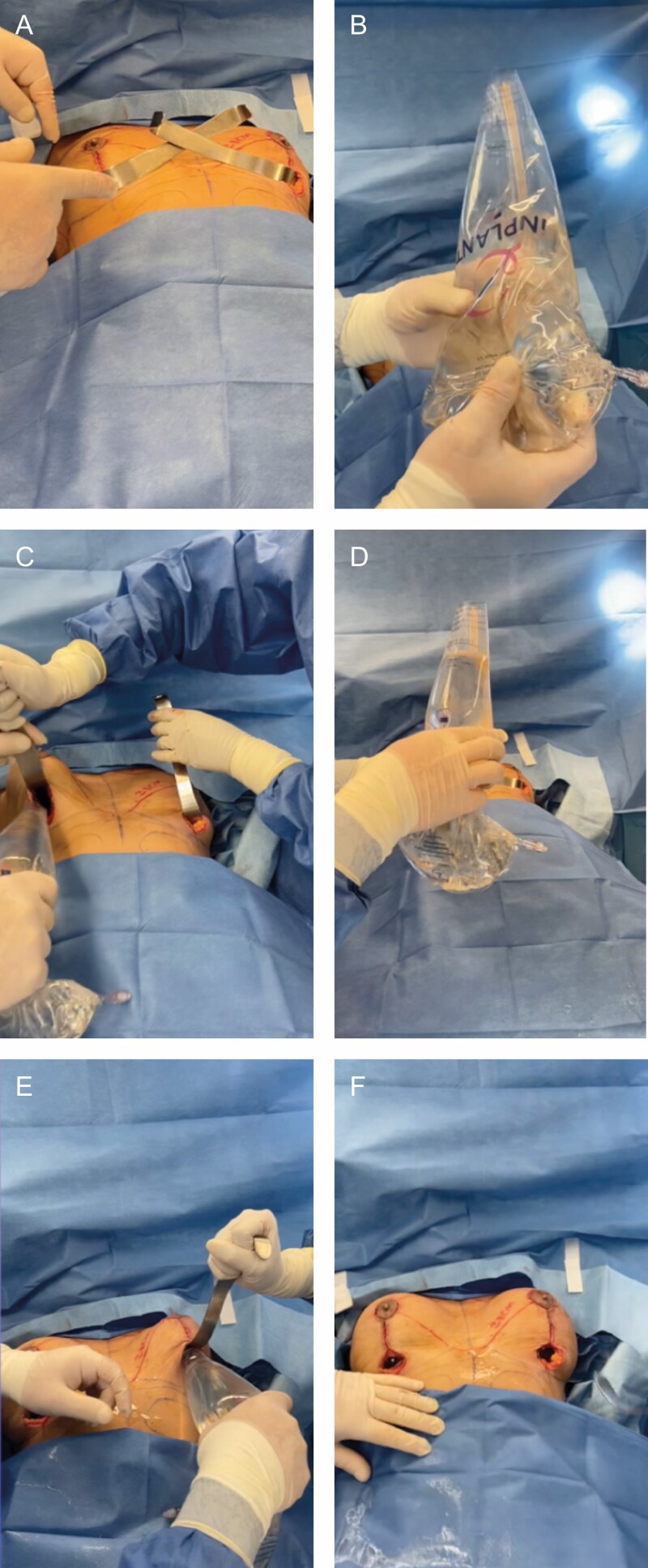
A 59-year-old female undergoing combined breast augmentation/mastopexy. (A) Pockets are dissected, and two retractors are placed in the wounds. (B) Two 450cc implants are loaded into the funnel; the tip is not cut. (C) Implant number one is inserted. (D) Funnel is ready to insert implant number two. (E) In rapid succession (17 seconds after the placement of implant number one) implant number two is inserted. (F) In 24 seconds, insertion of both implants is completed. Images are courtesy of iNPLANT LLC (Proximate Concepts LLC, Allendale, NJ, USA).

According to The Aesthetic Society’s annual statistics report, breast augmentation (BA) procedures continually rank among the most performed procedures, with a total of 280,692 performed in 2019 by 87.3% of plastic surgeons certified by the American Board of Plastic Surgery.^1^ There is a paucity of data in the plastic surgery literature relating to the use of funnels during BA and breast reconstruction procedures, even though protective funnels and other delivery devices are used commonly and considered a safe option with an average or below average infection and complication rate (Lombardo et al, unpublished data, February 2021). The authors present a double breast implant loading technique using the iNPLANT Funnel (Proximate Concepts LLC, Allendale, NJ, USA) that offers an innovative option for plastic surgeons versus the traditional two-hand manual insertion technique.^2^ Video 1 and Figure 1 show the double-loading technique where two implants are deployed in rapid succession within 24 seconds. Additional benefits of the procedure are described in this article and may encourage plastic surgeons to consider adopting protective funnels it in their practices.

Using a protective funnel to perform aesthetic and reconstructive breast procedures can reduce time in the operating room (OR) and a surgeon’s physical contact with the implants prior to insertion—often referred to as a “no touch” or “minimal touch” technique. The iNPLANT Funnel is a US Food and Drug Administration (FDA) Class 1 device with a lubricious hydrophilic coating on the inside surface. It is a closed system with the proximal end sealed, which permits the implant to rest in whatever antimicrobial solution is inserted into the funnel by the surgeon. This “bath” provides a secure and isolated environment inside the funnel for a longer time before delivery into the breast cavity.

With both pockets dissected, use of the double-loading technique allows for breast implants to be delivered in a short period of time. Notably, the first implant loaded into the iNPLANT funnel is the second be delivered and surgeons should bear this in mind. This sequence is crucial, especially when implants of different sizes are being implanted into the patient. The iNPLANT funnel is loaded by opening a seal (GlideTrack, patent pending, Proximate Concepts LLC, Allendale, NJ, USA) along the length of the device and placing the implant into the funnel through this opening. The first implant loaded will fall gravitationally to the wider base of the funnel. The second implant loaded will then fall gravitationally to the slightly narrower portion of the funnel because of its tapered shape. With the GlideTrack then resealed, the second loaded implant rests closest to the distal opening of the funnel and therefore is the first to exit and be delivered. This contrasts with the method of loading the Keller Funnel (Allergan, Dublin, Ireland), where the top (wide) portion of the funnel is open and the implant is loaded in a distal-to-proximal direction. This technique saves the surgeon excess motion in the operating room enabling less exposure to the ambient environment (eg, bacteria). The time to load both implants into the protective funnel is nominal. The primary benefit is a single preparation and loading of the funnel for both sides, along with the possible need to add more antimicrobial fluid or Betadine and opening and closing the GlideTrack to deliver the second implant. The use of a closed-system funnel can also reduce the likelihood that an implant may fall out, which would add additional time, cost, and potential for complications. Use of this technique has clinical applications in all surgical settings from hospitals to private practices and accredited outpatient surgical facilities. There is no cost to the surgeon who is already using a protective funnel and it may decrease the need for additional anesthesia.

Since the seal patch on breast implants is placed posteriorly (directly in contact with the chest wall) the surgeon should be mindful of the positional orientation of the implant (ie, location of this patch) as each implant is being prepared for delivery, crowned, and passed through the translucent funnel. Therefore, with the GlideTrack facing down, the seal patch on the implant should also be facing down and visible beneath the GlideTrack. If it is not, the surgeon can flip the implant within the funnel and re-orient it. Unintentional flipping can be avoided by careful placement of the implant into the funnel such that the seal patch is visible in the proper position, especially for smaller-sized implants.

The origin of the idea for the double loading technique was conceived when the author (PR) was approached by one of the major US-based implant manufacturers who inquired whether the iNPLANT Funnel could accommodate implants ranging from 800 cc to 1200 cc. The company was performing research to develop an implant for the transgender community and a competitive funnel could not accommodate such a large implant because it is open-ended. The author tested the iNPLANT Funnel, recorded it on video, and found that it could accommodate 3 implants with a total volume of 900 cc to 1200 cc. The author (PR) first performed the double loading technique in July 2018 and is not aware of prior presentations or published articles where a closed-system funnel was used to double load breast implants. The authors therefore believe this to be the first article in the literature showing the use of the technique.

Flugstad et al showed that the use of a funnel makes insertion easier for the surgeon, reduces trauma to implants during insertion, lessens contact with surgeons’ hands or skin and therefore reduces the risk for potential contamination and capsular contracture (CC).^3^ Studies have also shown that the use of a protective funnel to prevent the contamination of breast implants can yield reductions in the incidence of CC and up to a 50% reduction in reoperation rates due to CC.^2,4^

This technique elucidates the importance of considering innovative options during breast augmentation or reconstruction procedures. The double loading procedure can be implemented easily in plastic surgery practices and the authors hope the videos provide an educational example of the technique that can be adopted by surgeons who use protective funnels. Informed consent was obtained by the authors and the principles of the Declaration of Helsinki were followed. Institutional Review Board approval was not required for this study.

There are limitations to this article, as the author did not compare outcomes to cases where another funnel was used or where the double-loading procedure was not used. Therefore, the author cannot attest to efficacy of the technique in devices other than the iNPLANT Funnel. The goal of this article is to offer an alternative approach that minimizes the time to implant insertion and to highlight the potential benefits to plastic surgeons with educational videos in English (Video 2) and Spanish (Video 3) to assist those who wish to learn and implement the technique or adopt use of a protective funnel.
